# Managing Psychosis in Acute Intermittent Porphyria: A Case Report on Olanzapine Use

**DOI:** 10.7759/cureus.85815

**Published:** 2025-06-11

**Authors:** Chwee Fern Ooi, Chao Tian Tang, Sing Qin Ting, Iris Rawtaer, Ho Teck Tan

**Affiliations:** 1 Department of Psychiatry, Sengkang General Hospital, Singapore, SGP

**Keywords:** acute intermittent porphyria (aip), antipsychotics, neuropsychiatric symptoms, olanzapine, psychosis

## Abstract

Acute intermittent porphyria (AIP) is the most common type of acute porphyria, caused by reduced porphobilinogen deaminase activity, leading to accumulations of neurotoxic compounds. Symptoms usually include abdominal pain, autonomic dysfunction, neurological involvement, and psychiatric symptoms. Neuropsychiatric symptoms such as psychosis are common and may contribute to diagnostic delays. We report a case of a middle-aged woman presenting with psychiatric and neurovisceral symptoms of AIP.

This report discusses the case of a 41-year-old woman with no prior psychiatric history presenting with an acute onset of psychotic symptoms, including persecutory, erotomanic, and Capgras delusions, disorganized behavior, and agitation, along with physical symptoms of acute abdominal pain and autonomic disturbances. An extensive organic workup revealed elevated urinary porphobilinogen, which confirmed the diagnosis of AIP. The patient was treated medically, and her psychiatric symptoms completely resolved with olanzapine within eight days, without adverse effects.

This case reveals the importance of considering AIP in patients presenting with acute psychosis, particularly when accompanied by autonomic and abdominal symptoms. Olanzapine appears to be a safe and effective treatment for AIP-related psychosis, though careful monitoring is essential due to the variable drug responses and potential hepatic risks. Further studies are needed to establish standardized treatment guidelines for neuropsychiatric symptoms in AIP.

## Introduction

Acute porphyrias are a rare group of metabolic disorders caused by enzymatic defects in the heme biosynthesis pathway, resulting in toxic metabolite buildup [1,2]. Acute intermittent porphyria (AIP) is the most common form of acute porphyria, with a prevalence of five to ten individuals per 100,000 [3] and one in 2,000 across Europe. However, in Sweden, the incidence is higher, affecting approximately one in 1,000 individuals due to the founder effect [4].

AIP is inherited in an autosomal dominant pattern with variable expressivity and results from mutations that decrease the activity of porphobilinogen deaminase, also known as hydroxymethylbilane synthase. This enzyme deficiency leads to the accumulation of toxic compounds such as porphobilinogen (PBG) and δ-aminolevulinic acid (ALA), which causes neurological damage [2,5], manifesting as peripheral or autonomic neuropathies and psychiatric manifestations [6].

AIP manifests with a broad range of symptoms, often causing diagnostic delays [7]. It disproportionately affects women, with a female-to-male ratio of 1.5 to 2:1, and typically manifests between ages 18 and 40 [6].

This report presents a case of AIP with acute neurovisceral and psychiatric manifestations. Notably, the patient responded well to olanzapine, achieving rapid symptom resolution without signs of liver dysfunction, highlighting a potential role for this atypical antipsychotic in managing AIP-associated psychosis.

## Case presentation

A 41-year-old Chinese woman with no prior medical or psychiatric history presented with a one-week history of abrupt behavioral changes. She presented with persecutory, erotomanic, and Capgras delusions involving her parents. She believed that her colleagues and parents were trying to harm her and was convinced that she was in a romantic relationship with a male colleague. Additionally, she held the fixed belief that her parents were not her biological parents and had been replaced by impostors. She denied experiencing auditory or visual hallucinations, thought interference, or passivity symptoms. There was no significant decline in her daily functioning; she worked as an engineer and had been performing well at work before symptom onset. These psychiatric issues were preceded by transient, nonspecific abdominal pain several months earlier, which had resolved spontaneously.

The patient was born in China and relocated to Singapore in her 20s. She reported no family history of mental illness but described childhood adversity, including physical punishment by her mother at the age of seven due to academic difficulties. She denied alcohol or substance use, recent lifestyle changes, significant psychosocial stressors, or any dietary modification, such as dieting or reduced carbohydrate intake, before the onset of her illness. Her premorbid personality was described as introverted and calm. There was no family history of autoimmune diseases.

During hospitalization, she developed recurrent abdominal pain and fever. Her paranoia intensified, leading to her food refusal. She became increasingly restless, disorganized, and irritable, resulting in episodes of agitation, during which she attempted to dash out of her ward cubicle and climb out of bed. These behaviors necessitated the use of rapid tranquillization (oral and intramuscular lorazepam) and brief physical restraints to ensure her safety and that of others.

Physical examination revealed no overt neurological deficits or other abnormalities. However, she had episodes of low-grade fever (ranging from 37.6°C to 37.9°C) and tachycardia (up to 114 bpm) but no hypertension.

Given the acute onset of psychotic symptoms alongside physical and autonomic disturbances with tachycardia and fever, exclusion of organic causes was prioritized. Brief psychotic disorder was considered in the differential diagnosis based on Diagnostic and Statistical Manual of Mental Disorders, Fifth Edition criteria, given the presence of delusions and disorganized behavior lasting more than one day but less than a month (approximately one week for this case). The diagnosis was further supported by the absence of mood symptoms or substance use. However, investigations for underlying medical conditions were still ongoing at that time.

**Figure 1:**
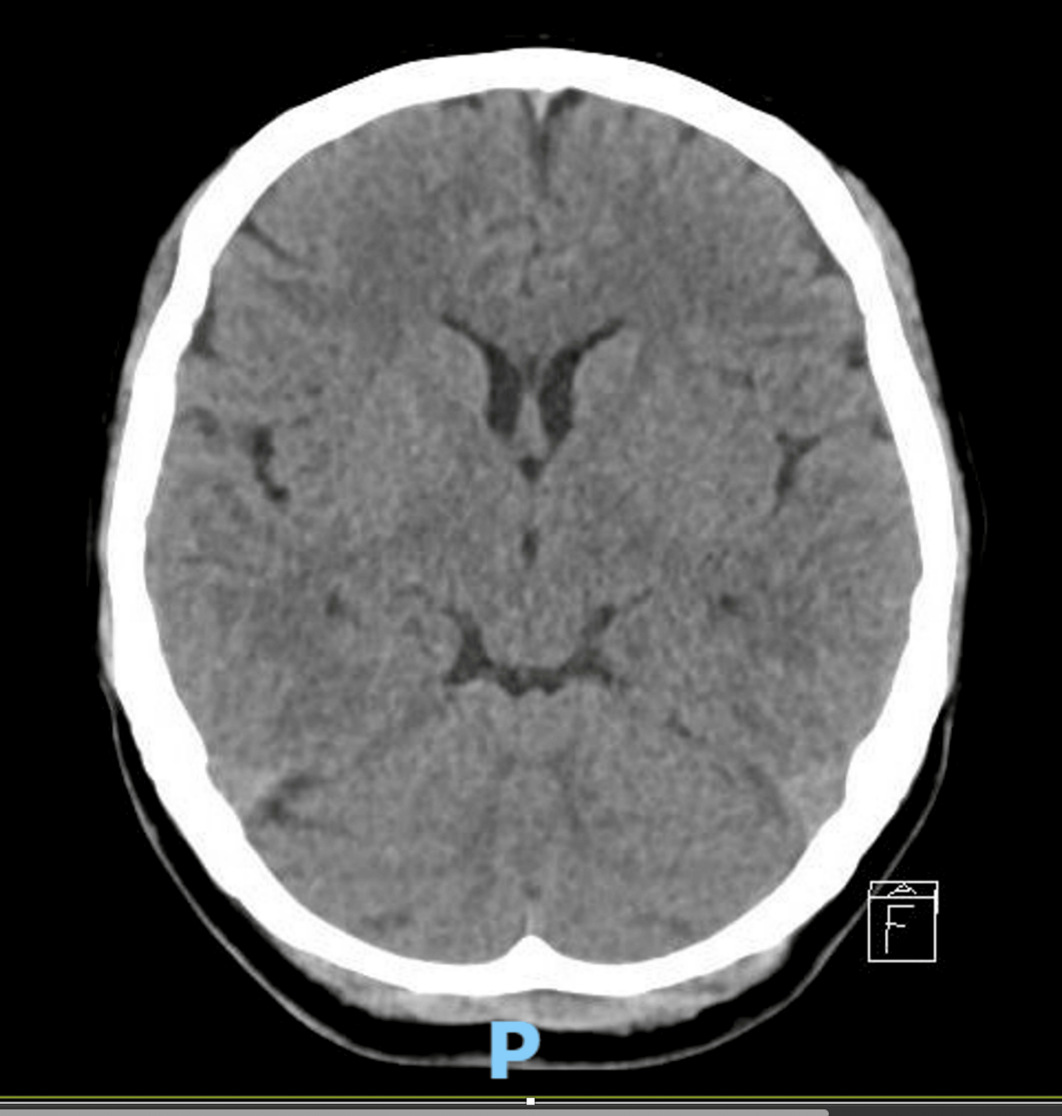
Noncontrasted CT brain revealed no evidence of acute intracranial abnormalities such as infarct, hemorrhage, or mass effect. The ventricular system appeared normal in size and configuration CT: computed tomography

**Figure 2:**
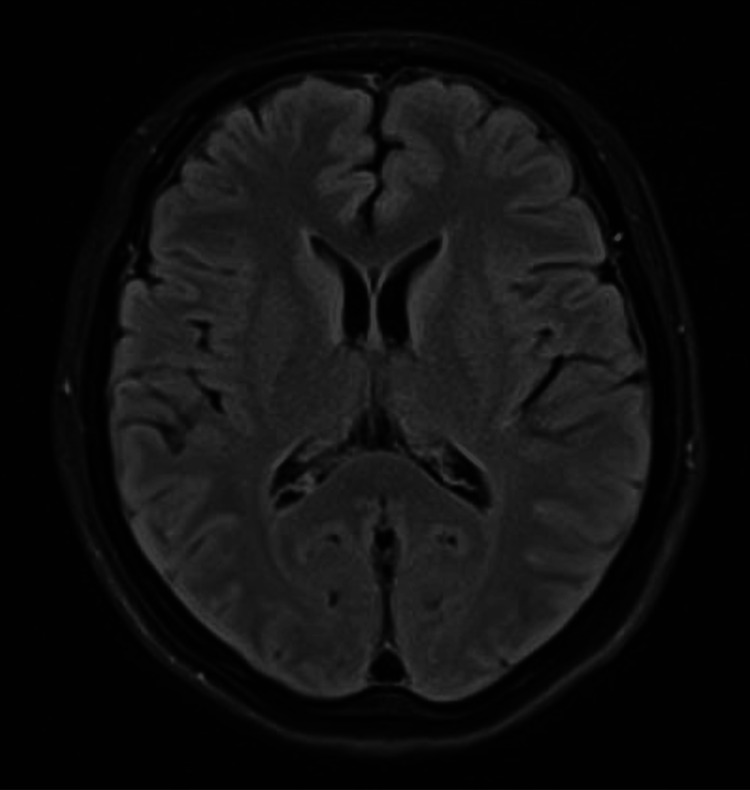
Contrasted MRI brain showed no evidence of intracranial pathology, such as infarct, hemorrhage, mass lesion, or parenchymal enhancing lesion. The ventricles, sulci, and cisterns are age-appropriate in size and configuration. The basal cisterns are preserved MRI: magnetic resonance imaging

Basic laboratory tests, including complete blood count, renal and liver function, thyroid and pregnancy tests, infectious screening, and urinalysis, showed no significant abnormalities or evidence of infection (Tables 1, 2). The toxicology screen was negative (Table 1). Brain imaging studies, including both a computed tomography scan (Figure 1) and a contrast-enhanced magnetic resonance imaging (Figure 2), were unremarkable. An EEG showed normal findings. Additionally, a lumbar puncture was performed, revealing normal cerebrospinal fluid analysis (Table 3). Tests for paraneoplastic antibodies, autoimmune encephalitis, and autoimmune panel were also negative (Tables 4-6).

**Table 1:** Comprehensive blood tests were within normal range, and toxicology screen was negative for all tested substances WBC: white blood cell; T4: thyroxine; Beta-hCG: beta-human chorionic gonadotropin; HIV: human immunodeficiency viruses; VDRL: venereal disease research laboratory; MDMA: 3,4-methylenedioxymethamphetamine; LSD: lysergic acid diethylamide

Parameters	Result	Reference range
Hemoglobin	13.3	12-16 g/dL
WBC count	7.99	4-10 × 10^9^/L
Platelet count	304	140-440 × 10^9^/L
Urea	3.3	2.5-7.8 mmol/L
Sodium	138	133-146 mmol/L
Potassium	4.0	3.5-5.1 mmol/L
Creatinine	44	45-84 umol/L
Glucose	5.9	3.9-11.0 mmol/L
Magnesium	0.87	0.7-1.0 mmol/L
Corrected calcium	2.18	2.1-2.6 mmol/L
Inorganic phosphate	1.30	0.8-1.5 mmol/L
Free T4	17.8	11.9-21.6 pmol/L
Thyroid-stimulating hormone	2.05	0.270-4.200 mIU/L
Total protein	61	68-85 g/L
Albumin	40	35-50 g/L
Total bilirubin	9	≤21 umol/L
Alkaline phosphatase	51	35-104 U/L
Alanine transaminase	21	≤35 umol/L
Aspartate transaminase	21	≤35 umol/L
Beta-hCG	<0.6	≤5.0 IU/L: nonpregnant
Lactate	0.9	0.5-2.2 mmol/L
Ammonia	14	1.6-53 umol/L
C-reactive protein	<0.6	≤4.9 mg/L
HIV	Nonreactive	Nonreactive
VDRL (syphilis)	Nonreactive	Nonreactive
Opioids	Negative	Negative
Tricyclic antidepressants	Negative	Negative
Amphetamine	Negative	Negative
Methamphetamine	Negative	Negative
Ketamine	Negative	Negative
MDMA	Negative	Negative
LSD	Negative	Negative

**Table 2:** Urinalysis showed mild abnormalities with trace ketonuria with raised red blood cell counts. These findings were nonspecific and not clinically significant in the absence of urinary symptoms RBC: red blood cell; WBC: white blood cell

Parameter	Results	Reference range
Glucose	Negative	Negative
Ketones	1+	Negative
RBC	25	0-3/uL
WBC	5	0-6/uL
Epithelial cells	0	0-4/uL
Cast, crystals, microorganisms	Negative	Negative
Bilirubin	Negative	Negative
Specific gravity	1.015	1.02-1.035
pH	6.5	4.6-8.0
Protein	Negative	Negative
Urobilinogen	1.0	0.2-1.0 EU/dL
Nitrite, leucocytes	Negative	Negative

**Table 3:** Cerebrospinal fluid analysis showing normal results WBC: white blood cell; RBC: red blood cell; AFB: acid-fast bacilli; TB: tuberculosis; PCR: polymerase chain reaction

Parameter	Results	Reference range
Appearance	Colorless	Colorless
WBC count	0	0-5/uL
RBC count	0	0-5/uL
Glucose	3.2	2.2-3.9 mmol/L
Total protein	0.35	0.15-0.45 g/L
Cytology, culture	Negative	Negative
AFB, TB tests	Negative	Negative
Meningitis/encephalitis PCR	Negative	Negative

**Table 4:** Autoimmune encephalitis panel was tested negative NMDAR: N-methyl D-aspartate receptor; CASPR2: contactin-associated protein 2; AMPAR1/2: alpha-amino-3-hydroxy-5-methyl-4-isoxazolepropionic acid receptor 1 and 2; LGI1: leucine-rich glioma-inactivated protein 1; DPP6: dipeptidyl-peptidase-like protein-6; GABABR: gamma-aminobutyric acid receptor B

Antibody tests	Result
NMDAR	Negative
CASPR2	Negative
AMPAR1/2	Negative
LGI1	Negative
DPPX/DPP6	Negative
GABABR	Negative

**Table 5:** Paraneoplastic antibodies panel was negative CRMP: collapsin response mediator protein; PNMA2: paraneoplastic antigen Ma2; ANNA: antineuronal nuclear antibody; PCA: Purkinje cell autoantibody; SOX 1: SRY-like high mobility group box; Zic4: zinc-finger protein 4; GAD65: glutamic acid decarboxylase 65; DNER: delta/notch-like epidermal growth factor-related receptor

Antibodies tested	Result
Amphiphysin	Negative
CV2/CRMP5	Negative
PNMA2/Ta	Negative
Ri (ANNA-2)	Negative
Yo (PCA-1)	Negative
Hu (ANNA-1)	Negative
Recoverin	Negative
SOX1	Negative
Titin	Negative
Zic4	Negative
GAD65	Negative
Tr (DNER)	Negative

**Table 6:** Autoimmune panel was negative while urine porphobilinogen was tested positive IIF: indirect immunofluorescence; DNA: deoxyribonucleic acid; SSA: Sjögren's syndrome antigen A; SSB: Sjögren's syndrome antigen B

Parameters	Results	Reference range
Antineutrophil cytoplasmic antibody (IIF)	Negative	Negative
Anti-double-stranded DNA antibody	<0.6	<10 IU/mL: negative
Smith antibody, ribonucleoprotein antibody, Ro (SSA) antibody, La (SSB) antibody, Scl 70 antibody, Jo-1 antibody	<1.0	<1.0: negative
Urine porphobilinogen	Positive	Negative

The neurology team was consulted and recommended a thorough organic workup, including urine PBG testing as part of a broader workup for unexplained autonomic and abdominal symptoms, given clinical suspicion of a metabolic etiology such as AIP. This test subsequently yielded a positive result (Table 6).

Treatment was initiated with olanzapine orodispersible tablets at 5 mg nightly on the sixth day of admission due to persistent psychotic symptoms, prior to confirmation of a porphyria diagnosis. Within three days, the patient exhibited clinical improvement, becoming calmer, less confused, and more communicative.

Urine PBG testing was performed on the seventh day of admission, and a positive result was received on the 11th day of admission, confirming a diagnosis of AIP with neurovisceral and neuropsychiatric symptoms. In response, the hematologist recommended two days of intravenous dextrose saline, which was initiated on the same day. Concurrently, the patient was referred to a dietitian for carbohydrate loading. As she continued to improve with existing psychiatric and medical treatment, intravenous hemin was not administered. She was advised to avoid alcohol and hormonal medications, and genetic counseling was provided to her parents.

The dose of olanzapine was gradually increased to 10 mg nightly. By the 13th day of admission (the eighth day of antipsychotic treatment), she achieved complete resolution of psychotic symptoms. Her family noted a return to her baseline personality. Liver function tests were checked twice during treatment and remained normal throughout.

A follow-up urine porphyrin test on October 3, 2024, was normal. At discharge, she reported no residual psychotic symptoms and was discharged into the care of her family. Follow-up appointments with psychiatry and hematology were scheduled for ongoing monitoring.

## Discussion

Acute AIP attacks are typically triggered by factors such as medications, infections, alcohol use, steroid hormones, and dietary factors such as fasting or reduced carbohydrate intake. AIP affects women more frequently than men. Symptoms typically manifest postpuberty, between the ages of 18 and 40 years [6]. Sex hormones like estrogen and progesterone are thought to increase ALA synthase activity, leading to a rise in porphyrin precursors [8]. Notably, these findings align with our case, as the patient is a woman within the expected age range.

Acute attacks typically present with severe abdominal pain, gastrointestinal symptoms (nausea and constipation), autonomic dysfunction (palpitations, tachycardia, sweating, and hypertension), and neurological manifestations such as confusion, peripheral neuropathy, paresis, or seizure [7]. Acute abdominal pain is the most common cause of admission in acute porphyria [9]. Patients may also report urine darkening to a reddish hue, especially upon light exposure [1]. Similarly, our patient experienced transient, nonspecific abdominal pain before presentation, a common initial symptom, accompanied by tachycardia and hypertension.

Psychiatric manifestations occur in 24%-80% of AIP cases. Psychiatric symptoms such as psychosis, anxiety, depression, and agitation further complicate diagnosis [1,10]. Depression and delirium were the most frequent neuropsychiatric manifestations [11]. In our case report, our patient presented with psychosis (with persecutory, erotomanic, and Capgras delusions) with episodes of agitation, which is in line with the common psychiatric manifestations of AIP.

According to the drugs safe list produced by Welsh Medicines Information Centre and Cardiff Porphyria Service in 2014 [12] and is supported by the National Acute Porphyria Service, the antipsychotics that are considered to be safe for use in the acute porphyria are haloperidol, chlorpromazine, fluphenazine trifluoperazine, amisulpiride, sulpiride, clozapine, and olanzapine. Meanwhile, chlorpromazine and trifluoperazine are typically recommended for use in porphyria, holding the longest record of safe use in this illness (Table 7).

**Table 7:** Four published case reports document the use of olanzapine in AIP, with mixed outcomes AIP: acute intermittent porphyria; ALT: alanine transaminase; AST: aspartate transaminase

Study	Treatment	Outcome	Side effects
Holroyd and Seward [13]	Olanzapine, trifluoperazine, and risperidone	Poor response	Trifluoperazine: drug-induced Parkinsonism
Bautista et al. [14]	Olanzapine, aripiprazole, and clozapine	Poor response	Aripiprazole: akathisia olanzapine: muscle cramping
Horgan and Jones [15]	Olanzapine	Good response	Elevated liver enzymes (ALT/AST)
Strauss and DiMartini [16]	Olanzapine	Good response	None reported

The variability in response to antipsychotics in AIP remains an area of clinical uncertainty, with reported cases demonstrating mixed efficacy. Olanzapine, which was effective in our patient, has previously been associated with positive outcomes [15,16] but has also been linked with liver enzyme elevation [15] and poor symptom control [13,14]. One possible explanation is variability in drug metabolism, as olanzapine is primarily metabolized by CYP1A2 and CYP2D6 [17], enzymes that may be impacted by hepatic dysfunction in AIP. Patients with altered CYP1A2 activity may experience reduced clearance of olanzapine, leading to increased side effects or treatment failure.

In our case report, olanzapine was chosen for its efficacy in treating positive symptoms, favorable tolerability, and low risk of extrapyramidal side effects. It was also considered appropriate given the absence of preexisting metabolic conditions in this patient. While olanzapine is associated with potential metabolic side effects such as weight gain and impaired glucose tolerance [18], the patient did not exhibit any metabolic derangements during the course of treatment. The patient showed rapid improvement in the resolution of psychotic symptoms within eight days of psychiatric treatment. In contrast to the case reported by Horgan and Jones, our patient has shown stability in liver enzymes after treatment, providing further evidence for the safe use of olanzapine in acute porphyria.

Given the limited evidence and mixed reported case outcomes, careful selection of antipsychotics in AIP should consider both efficacy and safety. While olanzapine was well-tolerated in our case, clinicians should monitor for hepatic effects and treatment response on an individual basis. Future studies should investigate the role of CYP polymorphisms and receptor-binding properties in antipsychotic selection for AIP-associated psychosis.

This case highlights the complexity of managing psychiatric symptoms in AIP and emphasizes the importance of early recognition of symptoms and individualized treatment strategies. The decision to test for urinary PBG was crucial in clinching the diagnosis, particularly as AIP often presents with a mix of psychiatric and neurovisceral symptoms that can mimic primary psychiatric disorders. In patients presenting with acute psychosis alongside abdominal pain or autonomic instability, screening for porphyria, especially AIP, should be part of the differential workup. While olanzapine demonstrated good efficacy and tolerability in this patient, variability in drug response remains a challenge. Future research should focus on refining antipsychotic selection based on pharmacogenetic factors and long-term safety data. These insights are essential for developing standardized guidelines for the treatment of AIP-related neuropsychiatric manifestations.

## Conclusions

This case highlights the diagnostic and therapeutic challenges of neuropsychiatric symptoms in AIP. It was essential to consider AIP in patients with acute psychosis and coexisting autonomic or visceral symptoms. Prompt urine PBG can aid early diagnosis and appropriate management, potentially averting complications and reducing diagnostic delay. The patient’s positive response to olanzapine supports its potential safety and efficacy in AIP-associated psychosis. However, given mixed responses and reports of hepatic adverse effects in other cases, clinicians should monitor liver function closely when prescribing olanzapine for AIP. Further research is needed to establish standardized treatment guidelines, assess long-term safety, and investigate the role of genetic factors (e.g., CYP enzyme polymorphisms) in antipsychotic metabolism and efficacy in AIP.
